# The role of gender, work family conflict, and gender role attitudes in daily parental wellbeing

**DOI:** 10.1038/s41598-026-48583-3

**Published:** 2026-06-04

**Authors:** Laura Altweck, Silke Schmidt, Samuel Tomczyk

**Affiliations:** 1 Department Health and Prevention, Institute of Psychology, University of Greifswald, Greifswald, Germany; 2 German Center for Child and Adolescent Health (DZKJ), Partner Site Greifswald/Rostock, Greifswald, Germany

**Keywords:** Subjective well-being, Gender role attitudes, Work-family conflict, Ecological momentary assessment, Gender, Parents, Public health, Quality of life, Human behaviour

## Abstract

A typical day or week looks very different for modern fathers and mothers, where women still fulfil more family responsibilities. While, gender differences in subjective well-being are widely documented, gender is rarely considered to explain fluctuations in daily well-being. We addressed this gap while also considering related concepts like the work-family conflict and gender role attitudes. Participants (individual parents) completed a baseline questionnaire covering sociodemographic background, work-family conflict, and gender role attitudes. Additionally, they filled out daily well-being surveys four times a day over a one-week period. Sixty-eight participants (75% women, age: *M* = 37.6 years) and 1352 datapoints from the daily surveys were analysed using multilevel modelling. Daily well-being increased over the survey period and women reported better well-being compared to men. More liberal private gender role attitudes were related to better cognitive well-being. Greater work-family conflict, irrespective of direction, was related to lower daily well-being. Gender and particularly related frameworks like the work-family conflict are important explanatory factors in not only overall but also daily well-being.

**Supplementary Information:**

The online version contains supplementary material available at 10.1038/s41598-026-48583-3.

## Introduction

While subjective well-being tends to be relatively stable across time, compared to stress or mood, it still exhibits significant daily and weekly fluctuations ^1,2^. These fluctuations are generally explained by various internal factors like arousal levels or mindfulness ^3^ or external factors like natural environments ^2^, yet gender differences are scarcely focused on. On average, a typical day and a typical week looks very different for modern mothers and fathers. Mothers are still more likely to be responsible for childcare ^4^ while the father’s employment and earning is prioritised ^5^. So, fluctuation in well-being is likely also influenced by daily routines and gender-specific challenges. Despite growing similarities in daily life ^6^, distinct differences persist, especially in family responsibilities and household chores, where women often bear a greater burden^7,8^. Given that traditional beliefs about gender roles and conflicting work and family responsibilities influence general well-being, it is crucial to investigate how these factors interact and influence daily well-being. This exploratory study focuses on parents with caregiving responsibilities, a group that faces unique challenges in balancing work and family demands.

Subjective well-being encompasses individuals’ assessments and evaluations of their own lives, involving both reflective cognitive judgments including overall life satisfaction and emotional reactions to daily experiences, including positive emotions (e.g., happiness, excitement) and negative emotions (e.g., upset, stress)^9,10^.

Research examining time use and well-being retrospectively for the past day has found that more psychological distress and physical symptoms are reported on days when stressors were experienced compared to stress-free days^11^. Men and women also differed in the types of stressors reported, e.g., for younger men this was interactions with co-workers, while for middle aged women this was network stressors. This approach also examines sequences of time use of the previous day in relation to well-being and finds a clear relationship between the two^12^. Möwisch et al. ^13^ for example found that people experienced more negative affect while working (anger/frustration & mourning/worries) and doing housework (anger/frustration/stress & boredom/loneliness) than in other activities. This body of research gives insights into the association between time use and well-being, with the limitation that both are assessed retrospectively, making reported well-being subject to recall bias and influenced by the specific situations participants remember^14^. Ecological momentary assessment (EMA) – as is used in the current study – enables the investigation of real-time variation in well-being^15^. Little research using EMA has examined daily fluctuations in well-being in non-clinical populations: Compared to mood, well-being appears somewhat more stable over time^3^, while variation is greater for positive and negative affect than for cognitive well-being^16^. Well-being tends to decline over the course of a day and similarly towards the end of the week^2^ . Akay and Martinssonfor ^1^ instance found the ‘blue’ Sunday effect – i.e., a dip in well-being – which was more pronounced in men than women. This begs the question as to whether this can be explained by differences in men’s and women’s daily and weekly routines.

Although there is a trend towards equivalence in men’s and women’s everyday lives, clear differences are still evident^6^. When looking at families with children, mothers, like fathers, now spend most of their day doing paid work, however, they still take on more family commitments ^8,17,18^. According to Coltrane ^19^, women’s share of housework is double that of men’s, with the largest gender differences observed among those with children of preschool-age ^20^. Beyond childcare, if a relative or close person is unwell, it also mainly falls to women to take care of them, although both male and female carers report worse mental health than non-carers^21,22^. Previous research has well established gender differences in daily activities and their impact on health and well-being ^7,8,17,18,23^ whereby certain activities – e.g., household cleaning and unpaid care work – that are consistently related to worse outcomes ^24,25^, are in turn more likely to be completed by women^ 18^.

Nevertheless, findings on gender differences in overall well-being remain mixed: Some studies report higher satisfaction and happiness in women compared to men ^26,27^, others report the opposite ^28^. These inconsistencies may partly reflect gendered differences in coping and resource processes. Women’s well-being may remain relatively stable despite greater caregiving and domestic burdens because they tend to mobilize stronger social support networks and emotion-focused coping strategies (Matud, ^61^, Ptacek, Smith, & Dodge, ^62^). Men, in contrast, may experience lower well-being when work demands impede their traditional provider role, consistent with research on role strain (Barnett & Hyde,^63^). We therefore propose the exploratory hypothesis:

**H1)** Being a woman is associated with better daily well-being. A concept to explain these gender differences in daily routines as well as consequences for well-being, are differing gender roles and role attitudes. Van der Horst ^29^ defines gender role attitudes as “[…] views held by individuals regarding the roles men and women should play in society” (p. 2451). These can be separated by the social context in which they are enacted, namely gender roles in the private domain (e.g., housework, informal care work) versus in the public domain (e.g., education, paid work, political activities) ^30^. The gender-specific values anchored in the roles are developed through cultural socialization in the family, school, and at work ^31^. Overall, men report more traditional gender role attitudes than women ^32,33^, likely because men stand to profit more than women from more traditional norms ^34^. The trend is going towards a more egalitarian society, while older age cohorts still hold more traditional attitudes than younger ones ^35,^Saunders and Kashubeck-West^36^ found that women’s psychological well-being is positively associated with a feminist identity, which includes a rejection of traditional gender roles and often an enactment of more advantageous behaviours (e.g., pursuing higher education, seeking better career opportunities, prioritizing self-care & personal development)^. ^Kulik^ 37^ also examined gender role attitudes in parents and found that mothers – as compared to fathers – reported more egalitarian views and these were in turn related to higher emotional well-being. Thus we propose the following hypotheses:

**H2)** More liberal gender role attitudes are associated with better daily well-being.

Another framework that is frequently cited in the literature to explain gender differences in daily activities and well-being is that of the work-family conflict ^38^. It describes the conflict between work and family as arising from an unhealthy balance between these domains, where a person is compelled to prioritize demands from one domain over the other. The conflict can arise from either domain to overlap into the other: Work to family conflict occurs when work commitments interfere with family life, such as through work overload, long or irregular hours, interpersonal conflicts at work, or lacking support from supervisors. Conversely, family to work conflict arises when home obligations interfere with work, such as the presence of young children, primary responsibility for childcare, or caring for elderly family members ^38^. Compared to childless individuals, parents report increased conflict between these domains, especially those with younger children ^39^. Approximately 50% of parents experience work-family conflict ^40^, although there appear to be few gender differences in the reported conflict^ 41^. A meta-analysis by Byron ^42^ showed that increasing work-family conflict, in either direction, is associated with worse well-being, yet a systematic review by Cavagnis et al. ^43^ found only three studies that examined work-family conflict in relation to well-being longitudinally. Thus, what is still understudied is whether the conflict between these domains has similar effects on daily well-being and in parent samples. We therefore propose the following hypothesis:

**H3)** Lower work-family conflict is associated with better daily well-being.

In sum, gender differences are evident in both everyday routines as well as in their consequences for well-being ^7,8,17,18,23^. Parents, in particular, experience a greater burden on their well-being due to additional commitments associated with childcare ^20,44^. Therefore, our study will focus on mothers and fathers. Indeed, studies have shown that women who try to equally balance the domains of work and family (e.g., working part-time while taking care of the children) report a higher burden compared to childless women and those working full-time ^7,18^. The present study will further examine the explanatory role of gender role attitudes and work-family conflict in relation to gender differences in daily well-being.

## Methodology

### Study design

This work is part of a pilot quasi-experimental, nonrandomized controlled trial using EMA ^45^. The main study consisted of an intervention group, which participated in online questionnaires, daily EMA surveys, and a subgroup that was invited to an interview, as well as a control group, which only participated in the online questionnaires. To answer the research questions of the present paper and because the control group did not participate in the daily EMA surveys, we will focus on the intervention group. For the full study design see the trial registration and study protocol ^45^.

In the intervention group, participants filled in an online questionnaire and, on the next day, participants began with the daily surveys, which they filled out on their personal smartphone. The daily surveys (each approximately 5–7 min) took place four-times per day (7:30am, 12 pm, 4:30 pm, 9 pm) over a period of seven days. Data from the first survey day was excluded to allow an acclimatization period for participants to become familiar with the survey.

### Sample and recruitment

Individuals with at least one child, who is under 18 years and living in the household were included. Only individuals over the age of 18 years, without foster children and without children with a chronic illness or disability were included, as these factors have been shown to further increase the burden of parenting ^46^. In order to complete the daily surveys, participants also had to have access to an Android smartphone.

Participants were recruited primarily from the northeast region of Germany using an opportunistic sample by distributing leaflets to relevant groups (e.g., parent groups, nurseries, schools). In an initial phone conversation, participants were informed about the study goals and methods, informed consent was collected, and, finally, the inclusion and exclusion criteria were checked. Participants in the intervention group were offered a monetary incentive of €35 for the completion of the baseline and follow-up questionnaires as well as at least 50% of the daily surveys, an additional €15 if they completed at least 80% of the daily surveys, and an additional €10 if they took part in the interview.

The minimum sample size for the intervention group was calculated using G*Power (version 3.1.9.6; ANOVA, repeated measures, within group, effect size [well-being] = 0.25, α = 0.05, power = 0.80, and correlation among repeated measures = 0.5), and the target sample size is 64 participants, see Altwecket al.^ 45^ for details. Although the final analyses used multilevel modeling, the ANOVA-based approach provides a reasonable approximation for power in small-scale EMA designs when ICCs are moderate. To assess robustness, a post hoc sensitivity analysis using the observed ICC values (0.42–0.71) indicated that the achieved sample (68 participants, 1352 assessments) had 80% power to detect within-person effects as small as β = 0.25 and between-person effects of β = 0.35. Thus, the study was sufficiently powered to detect medium-sized effects at both levels.

### Instruments

**Sociodemographic background** (baseline). The following variables were included: *Gender* (woman, man [reference]), *age* (in years), *education* [International Standard Classification of Education (ISCED)-97^47^; low & medium: level 0–4 [reference], high: level 5–6), *employment status* (full-time work [reference], part-time work, currently not working), *relationship status* (in relationship, not [reference]), *number of children under 14 years* (numeric), *children between 14–18 years* (yes, no [reference]), and *family time* (average hours per day spent on family commitments [e.g., childcare, family time, housework] on a regular work day and on a free day).

**Gender role attitudes** (trait-level) (baseline) were measured using the Scale of Gender Role Attitudes ^30^. Participants were asked “For each of the following statements on the role of men and women, please indicate how much you agree or disagree with this”. The scale has a subscale for the private (housework, care work, homemaker) (sample item: “A job is good, but what most women really want is a home and children”) and public (education, labour market, politics) (sample item: “When a mother goes out to paid work, her children suffer”) domain. Gender role attitudes were measured with three items for each subscale on a scale of 1 (*agree completely)* to 4 (*disagree completely)*. Higher values reflect more liberal beliefs. The reliability for private gender role attitudes was acceptable (α = 0.60, ω = 0.62) and good for public gender role attitudes (α = 0.79, ω = 0.82), which is similar to previous research using the German scale (α = [0.51-0.76])^48^. Of the sample 75% reported highly liberal values on the public gender role attitudes subscale. Due to this highly skewed distribution, this subscale was transformed into highly liberal (4) and low to moderately liberal (1–3).

**Work-family conflict **(trait-level) (baseline) was measured using the Work and Family Conflict Scale^49^. Participants were asked “Please indicate how much the following statements apply to you”. The scale consists of the subscales work to family conflict (sample item: “My family has to take a back seat because of my work”) and family to work conflict (sample item: “My work prevents me from spending enough time with my family”). The reliabilities for the work to family conflict (α = 0.78, ω = 0.79) and family to work conflict (α = 0.72, ω = 0.73) sub-scales were good. Work-family conflict was measured with 5 items for each subscale on a scale of 1 (*agree completely)* to 7 (*disagree completely)*, with higher values reflecting higher conflict.

**Daily well-being**. *Affective well-being *was assessed with the question “Please rate how well the following adjectives describe your feelings at the current time” with two items for positive affect (happy, relaxed) (reliability: ICC_between_ = 0.43, ICC_within_ = 0.58) and two item for negative affect (nervous, downbeat) (reliability: ICC_between_ = 0.61, ICC_within_ = 0.39) on a 7-point likert scale (0 [does not apply at all]—6 [applies completely])^50^. *Cognitive well-being* was assessed with the life satisfaction item of the WHO-QoL-8 (“How satisfied are you with your life at the moment?”) (reliability: ICC_between_ = 0.68, ICC_within_ = 0.32) on a 5-point likert scale (1 [very unsatisfied] – 5 [very satisfied])^51^. *Stress* was assessed with the four items of the global measure of perceived stress on a 5-point likert scale (0 [not at all] – 4 [very]) (reliability: ICC_between_ = 0.72, ICC_within_ = 0.28)^52^. An example item includes “How much do you currently feel …” “unable to influence important things in your life?”. In sum, higher values reflect better affective and cognitive well-being but greater stress.

**Survey time**. A counter of the completed daily surveys was created, which ranged from 1–24.

### Statistical analyses

In the study protocol we described the planned analytic plan: Variance-analytic and regressive models with repeated measures and multilevel design will be used to analyse within-person change in daily well-being within the 7-day measurement. See Altweck et al. ^45^ for details.

We analysed missing data patterns as well as compliance with the daily surveys. For distributions within the data we calculated mean values and standard deviations for continuous variables, and relative frequencies for categorical variables. Descriptive comparisons between men and women were made using *t*-tests and chi-square tests.

To accommodate participants’ getting used to the daily EMA surveys ^53,54^, data from the first survey day was excluded from the analyses. As a result, up to 24 data points per person (i.e., 4 surveys per day for 6 days) were included (see above).

To answer the research questions, multilevel modelling was used (via Stata version 18) with maximum likelihood estimation (the Stata code can be found in supplementary file 1). In a first step, null models of daily well-being were tested to inspect variance between different levels and to examine multilevel models. These models can be expressed as:

$${Y}_{\mathrm{ti}}\hspace{0.17em}={\beta }_{0}\hspace{0.17em}\hspace{0.17em}+{\mathrm{u}}_{0\mathrm{i}}\hspace{0.17em}\hspace{0.17em}+\hspace{0.17em}{\varepsilon }_{\mathrm{ti}} ,$$

where Y*ti* denotes the outcome for time point t nested within person i, u0i represents the person-specific random intercept, and εti the residual error.

In a second step, two-level random intercept models (level 1: assessments, level 2: persons) were estimated for each outcome (rI models). These models included confounding variables (age, education, employment status, partnership status, number of children, & family time), predictors (work-family conflict & gender role attitudes), and a time parameter (survey time), and can be written as:

$$\hspace{0.17em}{Y}_{\mathrm{ti}}={\beta }_{0}\hspace{0.17em}\hspace{0.17em}+{\hspace{0.17em}\beta }_{1}{\mathrm{time}}_{\mathrm{ti}}\hspace{0.17em}+{\beta }_{2}\hspace{0.17em}{\mathrm{X}}_{\mathrm{i}}\hspace{0.17em}+{\beta }_{3}\hspace{0.17em}{\mathrm{Z}}_{\mathrm{ti}\hspace{0.17em}}+\hspace{0.17em}{\mathrm{u}}_{0\mathrm{i}}\hspace{0.17em}+{\varepsilon }_{\mathrm{ti}}\hspace{0.17em} ,$$

where X_i_ denotes person-level covariates (e.g., gender, sociodemographics) and Z_ti_ time-varying predictors (e.g., work–family conflict).

In a third step, random intercept and random slope models for outcomes with predictors and random slope for gender (rIrS model) were run, allowing slopes to vary across individuals. Here the same approach was taken regarding the confounding variables and predictors. Specifically, models including a random slope for time (and, where specified, for gender) were fitted:

$$\hspace{0.17em}{Y}_{\mathrm{ti}}={\beta }_{0}\hspace{0.17em}\hspace{0.17em}+{\hspace{0.17em}\beta }_{1}{\mathrm{time}}_{\mathrm{ti}}\hspace{0.17em}+{\beta }_{2}\hspace{0.17em}{\mathrm{X}}_{\mathrm{i}}\hspace{0.17em}+{\beta }_{3}\hspace{0.17em}{\mathrm{Z}}_{\mathrm{ti}\hspace{0.17em}}+{\mathrm{u}}_{0i}+\hspace{0.17em}{\mathrm{u}}_{1\mathrm{i}}{time}_{ti}\hspace{0.17em}+{\varepsilon }_{\mathrm{ti}}\hspace{0.17em} ,$$

with (u_*0i*_, u_*1i*_)^T^∼*N*(0,Σ), where an unstructured covariance matrix was specified.

For models including interaction effects between gender and the time-varying predictors, the specification was extended as follows:

$${Y}_{\mathrm{ti}}={\beta }_{0}\hspace{0.17em}\hspace{0.17em}+{\hspace{0.17em}\beta }_{1}{\mathrm{time}}_{\mathrm{ti}}\hspace{0.17em}+{\beta }_{2}\hspace{0.17em}{\mathrm{X}}_{\mathrm{i}}\hspace{0.17em}+{\beta }_{3}\hspace{0.17em}{\mathrm{Z}}_{\mathrm{ti}\hspace{0.17em}}+{\beta }_{4}({\mathrm{gender}}_{i}\text{ x }{\mathrm{Z}}_{ti})+{\mathrm{u}}_{0i}+{\varepsilon }_{\mathrm{ti}}$$

Survey-based predictors were grand-mean centred to examine the association between differences in trait variables (e.g., gender role attitudes) with changes in daily assessments. Models were run separately for the four indicators of daily well-being, that is positive and negative affective well-being, cognitive well-being, and stress. Sensitivity analyses with models varying the stepwise inclusion of variables (step 1: predictor variables, step 2: sociodemographic variables and predictor variables) were conducted. We report intraclass correlation (ICC), Akaike Information Criterion (AIC), in addition to regression coefficients to describe model fit.

## Results

The final sample (*N* = 68) consisted of 17 men (25%) and 51 women (75%) and 1352 data points of the daily surveys (see Table 1 for the sample description). The average age was 37.6 years. The sample was highly educated (62%), mostly in a relationship (72%), and nearly equally divided across full-time, part-time, and no employment. On average, participants spent 8.9 h per day on family commitments. There were few significant differences between men and women, men were merely significantly older (men: *M* = 40.3; women: *M* = 36.8).

**Table 1:** Descriptive statistics of sample: total and by gender (*N* = 68).

		**Total sample**		**Women**		**Men**	
		*M / n*	*SD / %*	*M / n*	*SD / %*	*M / n*	*SD / %*
				51	75.0	17	25.0
Age (years)		37.6	6.1	**36.8**	**5.2**	**40.3**	**7.6**
Relationship	In relationship	49	72.1	35	68.6	14	82.4
	Other	19	27.9	16	31.4	3	17.6
Nr. children	under 14 years	1.7	0.8	1.7	0.8	1.8	0.8
	between 14–18 years	7	26.9	0.4	0.6	0.1	0.4
Education	Low / medium	26	38.3	22	43.1	4	23.5
	High	42	61.8	29	56.9	13	76.5
Employment	Full-time	24	35.3	15	29.4	9	52.9
	Part-time	16	23.5	13	25.5	3	17.6
	None	28	41.2	23	45.1	5	29.4
**Family time (daily hours)**		8.9	2.6	9.2	2.6	8.2	2.3
WFC^1^		4.3	1.0	3.8	1.1	3.8	0.9
FWC^1^		3.3	1.0	3.3	1.1	3.3	0.8
Public GRA^2^	Highly liberal	54	79.4	42	61.8	12	17.6
	Low to moderately liberal	14	20.6	9	13.2	5	7.4
Private GRA^2^		3.2	0.6	3.2	0.6	3.4	0.7

**Bolded**: *p* < .05; Relationship: Relationship status; Nr. children: number of children; Employment: Employment status; WFC: Work to family conflict, FWC: Family to work conflict, GRA: gender role attitudes; ^1^Work-family conflict was assessed using the Work and Family Conflict Scale ^49^,^2^Gender role attitudes were assessed using the Scale of Gender Role Attitudes ^30^.

There was no missing data in sociodemographic background, gender role attitudes, and work-family conflict; except average family time (3%). The average individual number of completed daily surveys was 23.1 [83%] (*SD* = 3.6), which is considered high ^53^. Men (*M* = 25.1 [90%], *SD* = 1.9) completed significantly more daily surveys than women (*M* = 22.4 [80%], *SD* = 3.8) and the latter also showed greater variance (*t*(54.9) = 3.9, *p* < 0.001). Results from correlation analyses between the main variables can be found in supplementary file 2 supplementary Tables 1–19, also separately by gender.

### Multilevel modelling

The results of multilevel models are presented in Table 2. The null models showed that intra-individual variation was moderate to high for all outcomes (ICC = [0.42, 0.71]). An improvement was seen for the rI models compared to the null models and a further improvement was seen for the rIrS models.

**Table 2:** Results of the multilevel model analyses.

Model:	Null			Random intercept																															Random intercept and random slope																				
Step:				1																		2													1												2								
				*b*						LB						UB						*b*					LB				UB				*b*				LB				UB				*b*				LB			UB	
**Positive affective well-being**																																																							
Time point				**.17**			[			**.12**			,			**.21**			]			**.20**			[		**.12**		,		**.28**		]		**.17**		[		**.12**		,		**.21**		]		**.19**		[		**.11**		,	**.28**	]
Gender				-.30			[			-.76			,			.17			]			.02			[		-.01		,		.06		]		-.30		[		-.76		,		.17		]		.03		[		-.01		,	.06	]
WFC ^1^				.24			[			-.10			,			.58			]			**-1.08**			[		**-1.59**		,		**-.56**		]		.24		[		-.10		,		.59		]		**-1.08**		[		**-1.60**		,	**-.56**	]
FWC ^1^				.19			[			-.14			,			.52			]			**-.91**			[		**-1.40**		,		**-.43**		]		.19		[		-.14		,		.52		]		**-.91**		[		**-1.41**		,	**-.42**	]
Private GRA ^2^				.17			[			-.17			,			.50			]			.20			[		-.18		,		.59		]		.17		[		-.17		,		.50		]		.20		[		-.19		,	.59	]
Public GRA ^2^				-.25			[			-.85			,			.35			]			.13			[		-.79		,		1.07		]		-.25		[		-.85		,		.35		]		.13		[		-.81		,	1.07	]
Age																						.53			[		-.07		,		1.12		]														.53		[		-.06		,	1.12	]
Education																						**.80**			[		**.14**		,		**1.46**		]														**.78**		[		**.11**		,	**1.44**	]
Nr. children under 14																						-.07			[		-.32		,		.19		]														-.07		[		-.33		,	.18	]
Nr. children 14–18																						**.55**			[		**.05**		,		**1.06**		]														**.55**		[		**.05**		,	**1.06**	]
Part-time employed																						**-1.40**			[		**-1.97**		,		**-.76**		]														**-1.36**		[		**-1.97**		,	**-.76**	]
Not employed																						.22			[		-.38		,		.82		]														.23		[		-.38		,	.83	]
Time with family																						**.70**			[		**.14**		,		**1.25**		]														**.70**		[		**.14**		,	**1.25**	]
ICC person	.42									.41																	.16												.42												< .01				
AIC	3846									3651																	1443												3645												1435				
Random slope variance (Gender)																																																			.07				
**Negative affective well-being**																																																							
Time point						**.08**			[			**.05**			,			**.12**			]			**.09**		[		**.02**		,		**.15**		]		**.08**		[		**.05**		,		**.12**		]		**.09**	[		**.02**		,	**.16**	]
Gender						-.36			[			-.86			,			.14			]			**.03**		[		**.00**		,		**.05**		]		-.36		[		-.86		,		.14		]		**.03**	[		**.01**		,	**.05**	]
WFC ^1^						.29			[			-.08			,			.66			]			**-.70**		[		**-1.10**		,		**-.30**		]		.29		[		-.08		,		.66		]		**-.73**	[		**-1.17**		,	**-.28**	]
FWC ^1^						-.05			[			-.40			,			.31			]			**-.88**		[		**-1.26**		,		**-.50**		]		-.05		[		-.40		,		.31		]		**-.86**	[		**-1.27**		,	**-.44**	]
Private GRA ^2^						-.00			[			-.36			,			.35			]			-.07		[		-.37		,		.23		]		-.00		[		-.36		,		.35		]		-.20	[		-.45		,	.05	]
Public GRA ^2^						-.55			[			-1.19			,			.09			]			-.50		[		-1.22		,		.22		]		-.55		[		-1.19		,		.09		]		-.52	[		-1.38		,	.33	]
Age																								-.14		[		-.60		,		.32		]														-.13	[		-.60		,	.33	]
Education																								**.90**		[		**.38**		,		**1.42**		]														.48	[		-.00		,	.97	]
Nr. children under 14																								.18		[		-.02		,		.38		]														.12	[		-.06		,	.29	]
Nr. children 14–18																								**.61**		[		**.22**		,		**1.00**		]														**.73**	[		**.29**		,	**1.16**	]
Part-time employed																								-.33		[		-.80		,		.14		]														-.29	[		-.74		,	.16	]
Not employed																								**.49**		[		**.02**		,		**.96**		]														.40	[		-.05		,	.85	]
Time with family																								**.84**		[		**.41**		,		**1.28**		]														**.82**	[		**.38**		,	**1.26**	]
ICC person			.60									.56																.13												.56											.19				
AIC			3294									3160																1304												3153											1291				
Random slope variance (Gender)																																																			.01				
**Stress**																																																							
Time point						-.02			[			-.04			,			.00			]			-.02		[		-.05		,		.01		]		-.02		[		-.04		,		.00		]		-.02		[		-.05	,	.01	]
Gender						.33			[			-.01			,			.66			]			-.02		[		-.05		,		.00		]		.33		[		-.01		,		.66		]		**-.04**		[		**-.06**	,	**-.02**	]
WFC ^1^						-.21			[			-.46			,			.04			]			**.82**		[		**.41**		,		**1.23**		]		-.21		[		-.46		,		.04		]		**.83**		[		**.35**	,	**1.30**	]
FWC ^1^						-.02			[			-.26			,			.22			]			**.79**		[		**.40**		,		**1.18**		]		-.02		[		-.26		,		.22		]		**.73**		[		**.30**	,	**1.16**	]
Private GRA ^2^						.02			[			-.22			,			.25			]			-.16		[		-.47		,		.15		]		.02		[		-.22		,		.25		]		**-.42**		[		**-.58**	,	**-.27**	]
Public GRA ^2^						.21			[			-.22			,			.64			]			-.52		[		-1.26		,		.22		]		.21		[		-.22		,		.64		]		-.25		[		-1.17	,	.67	]
Age																								-.04		[		-.52		,		.43		]														-.11		[		-.60	,	.38	]
Education																								**-.91**		[		**-1.44**		,		**-.37**		]														**-.54**		[		**-1.04**	,	**-.05**	]
Nr. children under 14																								-.00		[		-.20		,		.20		]														.07		[		-.08	,	.22	]
Nr. children 14–18																								**-.70**		**[**		**-1.10**		**,**		**-.30**		**]**														**-.75**		[		**-1.22**	,	**-.28**	]
Part-time employed																								**.89**		**[**		**.41**		**,**		**1.37**		**]**														**1.17**		[		**.76**	,	**1.59**	]
Not employed																								-.31		[		-.79		,		.17		]														-.26		[		-.69	,	.18	]
Time with family																								**-.60**		[		**-1.04**		,		**-.16**		]														**-.47**		[		**-.90**	,	**-.04**	]
ICC person			.71									.68																.48												.68												.29			
AIC			1557									1516																568												1510												547			
Random slope variance (Gender)																																																				< .01			
**Cognitive Well-Being**																																																							
Time point					**.04**			[			**.01**			,			**.07**			]			.04			[		-.00		,		.09		]		**.04**		[		**.01**		,		**.07**		]		.04		[		-.00	,	.09	]
Gender					-.20			[			-.62			,			.22			]			**.03**			[		**.00**		,		**.06**		]		-.20		[		-.62		,		.22		]		**.03**		[		**.00**	,	**.06**	]
WFC ^1^					.07			[			-.24			,			.38			]			**-.81**			[		**-1.29**		,		**-.34**		]		.07		[		-.24		,		.38		]		**-.81**		[		**-1.29**	,	**-.34**	]
FWC ^1^					.01			[			-.29			,			.31			]			**-.87**			[		**-1.32**		,		**-.42**		]		.01		[		-.29		,		.31		]		**-.87**		[		**-1.32**	,	**-.42**	]
Private GRA ^2^					.17			[			-.13			,			.47			]			**.56**			**[**		**.20**		**,**		**.92**		]		.17		[		-.13		,		.47		]		**.56**		[		**.20**	,	**.92**	]
Public GRA ^2^					-.18			[			-.72			,			.36			]			.69			[		-.17		,		1.55		]		-.18		[		-.72		,		.36		]		.69		[		-.17	,	1.54	]
Age																							.37			[		-.18		,		.92		]														.37		[		-.18	,	.92	]
Education																							**1.04**			[		**.43**		,		**1.65**		]														**1.04**		[		**.43**	,	**1.65**	]
Nr. children under 14																							**.37**			[		**.13**		,		**.60**		]														**.37**		[		**.13**	,	**.60**	]
Nr. children 14–18																							.19			[		-.27		,		.66		]														.19		[		-.27	,	.66	]
Part-time employed																							**-1.12**			[		**-1.68**		,		**-.57**		]														**-1.12**		[		**-1.68**	,	**-.57**	]
Not employed																							.16			[		-.39		,		.72		]														.16		[		-.39	,	.72	]
Time with family																							.35			[		-.16		,		.86		]														.35		[		-.16	,	.86	]
ICC person		.71									.67																	.35												.67												< .01			
AIC		1557									2188																	962												2182												956			
Random slope variance (Gender)																																																				< .01			

**Bolded**: *p* < .05; Nr. children: number of children; Employment: Employment status; WFC: Work to family conflict, FWC: Family to work conflict, GRA: gender role attitudes; ^1^Work-family conflict was assessed using the Work and Family Conflict Scale ^49^,^2^Gender role attitudes were assessed using the Scale of Gender Role Attitudes ^30^.

In the text we report significant effects (*p* < 0.05) of the models with control variables, all other effects were non-significant. In the rI models for affective well-being, an increase was seen over the survey time. The effect of survey time remained significant in the rIrS models. No change was seen in stress and life satisfaction.

*Gender (H1).* In the rI models, women showed better negative affective well-being (*b* = 0.03 [0.00, 0.05], *p* < 0.05) and cognitive well-being (*b* = 0.03 [0.00, 0.06], *p* < 0.05) compared to men. This effect remained significant in the rIrS models (negative affective well-being*: b* = 0.03 [0.01, 0.05], *p* < 0.05, cognitive well-being*: b* = 0.03 [0.00, 0.06], *p* < 0.05). Further, in the rIrS models, women also showed less stress compared to men (*b* = -0.04 [-0.06, -0.02], *p* < 0.05).

*Gender role attitudes (H2a)*. In the rI models, more liberal domestic gender role attitudes were related to higher life satisfaction (*b* = 0.56 [0.20, 0.92], *p* < 0.05). This held in the rIrS models (*b* = 0.56 [0.20, 0.92], *p* < 0.05) and they were also related to less stress (*b* = -0.42 [-0.58, -0.27], *p* < 0.05). No significant effects were seen for public gender role attitudes (*p* > 0.05).

*Work-family conflict (H3a)*. In both the rI and rIrS models, higher work to family conflict was significantly related to worse positive affective well-being (rI: *b* = -1.08 [-1.59, -0.56], *p* < 0.05; rIrS: *b* = -1.08 [-1.60, -0.56], *p* < 0.05), worse negative affective well-being (rI: *b* = -0.70 [-1.10, -0.30], *p* < 0.05; rIrS: *b* = -0.73 [-1.17, -0.28], *p* < 0.05), worse cognitive well-being (rI: *b* = -0.81 [-1.29, -0.34], *p* < 0.05; rIrS: *b* = -0.81 [-1.29, -0.34], *p* < 0.05), and less stress (rI: *b* = 0.82 [0.41, 1.23], *p* < 0.05; rIrS: *b* = 0.83 [0.35, 1.30], *p* < 0.05). Similarly, in both the rI and rIrS models, higher family to work conflict was significantly related to worse positive affective well-being (rI: *b* = -0.91[-1.40, -0.43], *p* < 0.05; rIrS: *b* = -0.91[-1.41, -0.42], *p* < 0.05), worse negative affective well-being (rI: *b* = -0.88 [-1.26, -0.50], *p* < 0.05; rIrS: *b* = -0.86 [-1.27, -0.44], *p* < 0.05), worse cognitive well-being (rI: *b* = -0.87 [-1.32, -0.42], *p* < 0.05; rIrS: *b* = -87 [-1.32, -0.42], *p* < 0.05), and less stress (rI: *b* = 0.79 [0.40, 1.18], *p* < 0.05; rIrS: *b* = 0.73 0.30, 1.16], *p* < 0.05).

*Sensitivity analyses and control variables*. We also ran models without control variables (Table 2). The predictor variables were not significantly associated with daily well-being in the models without control variables. The only exception was in the rIrS models without control variables, where women continued to report better negative affective well-being and lower stress than men. In addition, in the rIrS model without controls, women also reported higher positive affective well-being.

For brevity purposes we summarize the main findings regarding the control variables (see Table 2 for details): Age was not significantly related to daily well-being. Low or moderate versus high education was related to worse daily well-being across all outcomes. Similarly, part-time employment was associated with worse daily well-being compared to full-time employment, whereas no significant difference was seen for no employment. Having children under the age of 14 years was related to better cognitive well-being but having children of or over the age of 14 years was related to better affective wellbeing and less stress. Finally, greater time spent on family commitments was related to better affective well-being and less stress.

## Discussion

This study explored the daily well-being of mothers and fathers in relation to gender, work-family conflict, and gender role attitudes. In sum, women reported better affective well-being than men, yet other factors – i.e., work-family conflict – were found to be more relevant in explaining variations of daily well-being.

While the literature examining gender, related frameworks, and overall well-being is vast^ 26–28,55^, there is not as much knowledge about gendered state experiences of well-being. Studies examining fluctuations in daily well-being have generally drawn on internal factors like energy and arousal levels or mindfulness ^3^ or external factors like natural environments ^2^ and often neglect the effect of gender. With daily and weekly routines as well as tasks and responsibilities still being very different for modern men and women^ 7,17,18^, it is no surprise that our findings show that gender – and its associations – are associated with daily well-being. While the body of research examining gender differences in general well-being is vast, the findings are ambiguous ^26–28^. These mixed findings may arise because gender differences in well-being are highly contingent on contextual factors (e.g., distribution of paid and unpaid labour, caregiving intensity, daily role demands), which can differentially advantage or disadvantage individuals across settings and time scales. That being said, the current findings underscore women’s resilience, also when faced with carrying greater domestic and caregiving burdens.

In our study, the most relevant framework in explaining daily well-being across all outcomes was found to be that of the work-family conflict. In their meta-analysis ^41^ found that men and women did not report different levels of work-family conflict – which was also the case in our sample. We found that greater conflict between the domains of work and family, irrespective of the direction of the interference, was related to lower well-being. These effects were only seen once sociodemographic background – e.g., age, education and employment status – was controlled for. Given that the present sample was highly educated (62%), mostly in a relationship (72%), and reported highly liberal gender role attitudes, it is likely that the raw associations between work-family conflict and daily well-being were obscured by between group differences. Consistent with this notion Page et al. ^56^ showed that sociodemographic characteristics shape patterns of work–family conflict over time, indicating that such conflict is not merely an individual stressor but a structured, patterned experience. Individuals with more resources (e.g., higher education, stable employment, flexible work arrangements) may experience high work-family conflict yet show smaller drops in well-being, as these resources provide coping strategies and social support that buffer the impact ^57^. Controlling for sociodemographic factors therefore allows the underlying within-group association between work–family conflict and daily well-being to become more visible.

This is further supported by the finding that, compared to full-time employment, part-time employment was associated with lower daily well-being, whereas no employment did not differ significantly from full-time employment. This aligns with previous research showing that part-time roles are disproportionately taken by women ^7,18^, likely as a strategy to balance work and family demands. Yet such arrangements are also linked to worse general well-being in cross-sectional studies and across the life span ^7,18^. While part-time work arrangements are often intended to reduce strain, they may paradoxically sustain or even heighten work-family conflict. Part-time employees may still face high performance expectations at work while simultaneously carrying a substantial share of family responsibilities, resulting in pressure to excel in both domains without the time or resources to do so fully.

Additionally, more liberal private gender role attitudes were linked to better cognitive well-being. Liberal private gender role attitudes promote an equitable sharing of household and childcare responsibilities between partners. This balanced division of labour reduces the burden on any one individual, allowing greater satisfaction with life ^19,38^. Interestingly, this association was not observed for affective well-being or perceived stress, suggesting that liberal private gender role attitudes may contribute more to evaluative judgments of life quality than to day-to-day emotional states or immediate stress levels.

### Strengths, limitations, and future directions

A strength of the study is that it examined gender and related frameworks in relation to daily well-being, replicating existing literature focusing on state well-being ^26–28,55^. We focused on parents, as they experience greater burden on their well-being due to multiple, conflicting commitments between the domains of work and family ^20^. As such, a strength of the study was the recruitment of parents as a gender diverse group with lived experience of work-family conflict. In turn this focused sample may have implications for the generalizability of the results, for instance when considering other age groups that may have less pressures from multiple life domains. Another limitation is that the sample was highly educated and included few men. With a study focus on gender issues, there may innately be a sampling issue, less likely to attract men and, if so, attracting male ‘allies’ to participate. Indeed, the sample reported high to highly liberal gender role beliefs and we did not find differences between men and women. The current sample consisted exclusively of German participants. Given that gender role beliefs and norms can vary across cultural contexts ^58,59^, this may have influenced the findings. Since this is a pilot study, the inclusion criteria regarding parenthood were rather broad, and did not consider specific constellations (e.g., rainbow families, single-parent households) as subgroups that might provide further insight into parental stress and well-being, for instance, through an intersectional lens. We were also unable to examine dyadic-level couple factors – e.g., shared parenting responsibilities or co-habiting—which may influence parental well-being. Future studies should aim to recruit a wider, representative population samples and focus on parent-dyads to replicate the current findings. Although the current study assessed daily time use, our feasibility assessment revealed that parents reported confusion with daily time-use entries ^60^, so we were unable to include this aspect in the analysis. In future research this methodology should be further developed and also include other aspects like daily hassles.

## Conclusions

Our study illuminates the intricate relationship between work-family conflict and gender role beliefs in shaping the daily well-being of young and middle-aged women and men. It highlights that work-family conflict remains a crucial factor affecting well-being. Our findings call for tailored interventions that address the unique work-family conflict experiences and gender role belief tensions across genders to effectively support their well-being.

## Supplementary Information

Supplementary Information 1.

Supplementary Information 2.

## Data Availability

The data and analyses files that support the findings of this study are available on reasonable request from the corresponding author. The data are not publicly available due to privacy restrictions.

## Abbreviations

ISCED: International standard classification of education
rI models: Two-level random intercept models for outcomes
rIrS model: Random intercept and random slope models for outcomes with predictors and random slope for gender
AIC: Akaike information criterion
ICC: Intraclass correlations
