# Supplementary material for: The role of gender, work family conflict, and gender role attitudes in daily parental wellbeing
Source: Sci Rep. 2026 Jun 4;16:17356. doi: 10.1038/s41598-026-48583-3 (PMC13237153; doi:10.1038/s41598-026-48583-3)
Supplement: Supplementary file 1 — Supplementary Information 1. [file 41598_2026_48583_MOESM1_ESM.docx]

**Supplementary information 1.** Stata Code

**Prediction of affective well-being**

Positive affect predicted by gender, attitudes without interaction effects and with random intercept

mixed ema_affwb_pos timepoint i.sd01 c.wfc c.fwc [c.gr](http://c.gr)_d [c.gr](http://c.gr)_p||code:||timepoint:, cov(unstructured)

Positive affect predicted by gender, attitudes without interaction effects and with random intercept  and random slope

mixed ema_affwb_pos timepoint i.sd01 c.wfc c.fwc [c.gr](http://c.gr)_d [c.gr](http://c.gr)_p||code:||timepoint: i.sd01, cov(unstructured)

Positive affect predicted by gender, attitudes with interaction effects and with random intercept

mixed ema_affwb_pos timepoint i.sd01 c.wfc c.fwc [c.gr](http://c.gr)_d [c.gr](http://c.gr)_p  i.sd01#c.wfc i.sd01#c.fwc i.sd01#[c.gr](http://c.gr)_d i.sd01#[c.gr](http://c.gr)_p ||code:||timepoint:, cov(unstructured)

 Negative affect predicted by gender, attitudes without interaction effects and with random intercept

mixed ema_affwb_neg timepoint i.sd01 c.wfc c.fwc [c.gr](http://c.gr)_d [c.gr](http://c.gr)_p||code:||timepoint:, cov(unstructured)

Negative affect predicted by gender, attitudes without interaction effects and with random intercept  and random slope

mixed ema_affwb_neg timepoint i.sd01 c.wfc c.fwc [c.gr](http://c.gr)_d [c.gr](http://c.gr)_p||code:||timepoint: i.sd01, cov(unstructured)

Negative affect predicted by gender, attitudes with interaction effects and with random intercept

mixed ema_affwb_neg timepoint i.sd01 c.wfc c.fwc [c.gr](http://c.gr)_d [c.gr](http://c.gr)_p  i.sd01#c.wfc i.sd01#c.fwc i.sd01#[c.gr](http://c.gr)_d i.sd01#[c.gr](http://c.gr)_p ||code:||timepoint:, cov(unstructured)

**Prediction of stress**

Stress predicted by gender, attitudes without interaction effects and with random intercept

mixed ema_stress timepoint i.sd01 c.wfc c.fwc [c.gr](http://c.gr)_d [c.gr](http://c.gr)_p||code:||timepoint:, cov(unstructured)

Stress predicted by gender, attitudes without interaction effects and with random intercept  and random slope

mixed ema_stress timepoint i.sd01 c.wfc c.fwc [c.gr](http://c.gr)_d [c.gr](http://c.gr)_p||code:||timepoint: i.sd01, cov(unstructured)

Stress predicted by gender, attitudes with interaction effects and with random intercept

mixed ema_stress timepoint i.sd01 c.wfc c.fwc [c.gr](http://c.gr)_d [c.gr](http://c.gr)_p  i.sd01#c.wfc i.sd01#c.fwc i.sd01#[c.gr](http://c.gr)_d i.sd01#[c.gr](http://c.gr)_p ||code:||timepoint:, cov(unstructured)

**Prediction of life satisfaction**

Life satisfaction predicted by gender, attitudes without interaction effects and with random intercept

mixed ema_lz timepoint i.sd01 c.wfc c.fwc [c.gr](http://c.gr)_d [c.gr](http://c.gr)_p||code:||timepoint:, cov(unstructured)

Life satisfaction predicted by gender, attitudes without interaction effects and with random intercept  and random slope

mixed ema_lz timepoint i.sd01 c.wfc c.fwc [c.gr](http://c.gr)_d [c.gr](http://c.gr)_p||code:||timepoint: i.sd01, cov(unstructured)

Life satisfaction predicted by gender, attitudes with interaction effects and with random intercept

mixed ema_lz timepoint i.sd01 c.wfc c.fwc [c.gr](http://c.gr)_d [c.gr](http://c.gr)_p  i.sd01#c.wfc i.sd01#c.fwc i.sd01#[c.gr](http://c.gr)_d i.sd01#[c.gr](http://c.gr)_p ||code:||timepoint:, cov(unstructured)
