# Supplementary material for: The role of gender, work family conflict, and gender role attitudes in daily parental wellbeing
Source: Sci Rep. 2026 Jun 4;16:17356. doi: 10.1038/s41598-026-48583-3 (PMC13237153; doi:10.1038/s41598-026-48583-3)
Supplement: Supplementary file 2 — Supplementary Information 2. [file 41598_2026_48583_MOESM2_ESM.docx]

**Supplementary file 2.** Results from the correlation analyses

**Supplementary table 1**. Correlation effects for correlations of main variables

|  | sex | age | education | employment | relationship | no. children under 13 | no. children 14-18 | family time |
| --- | --- | --- | --- | --- | --- | --- | --- | --- |
| sex | 1 | -0.3120 | 0.2297 | 0.2265 | 0.1820 | 0.3030 | 0.6376 | 0.2593 |
| age | -0.3120 | 1 | -0.6732 | 0.0558 | -0.2556 | -0.0845 | 0.1816 | -0.0737 |
| education | 0.2297 | -0.6732 | 1 | 0.3291 | 0.1444 | 0.2900 | -0.5026 | 0.4151 |
| employment | 0.2265 | 0.0558 | 0.3291 | 1 | -0.2699 | 0.3704 | -0.3245 | 0.6318 |
| relationship | 0.1820 | -0.2556 | 0.1444 | -0.2699 | 1 | -0.4229 | 0.0382 | 0.0248 |
| no. children under 13 | 0.3030 | -0.0845 | 0.2900 | 0.3704 | -0.4229 | 1 | 0.1165 | 0.0807 |
| no. children 14-18 | 0.6376 | 0.1816 | -0.5026 | -0.3245 | 0.0382 | 0.1165 | 1 | -0.2672 |
| family time | 0.2593 | -0.0737 | 0.4151 | 0.6318 | 0.0248 | 0.0807 | -0.2672 | 1 |
| pos. Affect | 0.0852 | 0.0225 | 0.0005 | -0.0356 | 0.2228 | -0.0502 | 0.0731 | -0.1530 |
| negative affect | 0.0228 | 0.1797 | -0.1869 | 0.0908 | 0.1282 | 0.1414 | 0.1232 | -0.0015 |
| life satisf. | 0.1348 | -0.0625 | 0.1143 | 0.1024 | 0.0720 | 0.0526 | -0.0250 | -0.1143 |
| stress | 0.0338 | -0.1129 | 0.3175 | 0.0716 | -0.2179 | 0.1619 | -0.1517 | 0.2474 |
| GRA domestic | 0.0686 | -0.4609 | 0.2993 | -0.0657 | 0.5359 | -0.3143 | -0.2007 | 0.1022 |
| GRA public | -0.2300 | -0.1451 | -0.2085 | 0.1720 | -0.1973 | -0.3328 | -0.2502 | 0.0436 |
| WFC | 0.3992 | -0.0324 | -0.1769 | -0.0315 | 0.4647 | -0.1743 | 0.3032 | 0.2288 |
| FWC | -0.3337 | 0.1948 | -0.0229 | -0.1153 | -0.5401 | 0.2061 | -0.0396 | -0.3247 |

**Supplementary table 2**. Correlation effects for correlations of main variables (cont.)

|  | pos. Affect | negative affect | life satisf. | stress | GRA domestic | GRA public | WFC | FWC |
| --- | --- | --- | --- | --- | --- | --- | --- | --- |
| sex | 0.0852 | 0.0228 | 0.1348 | 0.0338 | 0.0686 | -0.2300 | 0.3992 | -0.3337 |
| age | 0.0225 | 0.1797 | -0.0625 | -0.1129 | -0.4609 | -0.1451 | -0.0324 | 0.1948 |
| education | 0.0005 | -0.1869 | 0.1143 | 0.3175 | 0.2993 | -0.2085 | -0.1769 | -0.0229 |
| employment | -0.0356 | 0.0908 | 0.1024 | 0.0716 | -0.0657 | 0.1720 | -0.0315 | -0.1153 |
| relationship | 0.2228 | 0.1282 | 0.0720 | -0.2179 | 0.5359 | -0.1973 | 0.4647 | -0.5401 |
| no. children under 13 | -0.0502 | 0.1414 | 0.0526 | 0.1619 | -0.3143 | -0.3328 | -0.1743 | 0.2061 |
| no. children 14-18 | 0.0731 | 0.1232 | -0.0250 | -0.1517 | -0.2007 | -0.2502 | 0.3032 | -0.0396 |
| family time | -0.1530 | -0.0015 | -0.1143 | 0.2474 | 0.1022 | 0.0436 | 0.2288 | -0.3247 |
| pos. Affect | 1 | 0.6331 | 0.6563 | -0.6022 | -0.0518 | -0.1392 | 0.0989 | -0.1176 |
| negative affect | 0.6331 | 1 | 0.4756 | -0.5723 | -0.1997 | -0.3303 | 0.2576 | -0.2137 |
| life satisf. | 0.6563 | 0.4756 | 1 | -0.5990 | 0.1498 | 0.0195 | 0.1518 | -0.2236 |
| stress | -0.6022 | -0.5723 | -0.5990 | 1 | 0.0422 | 0.0576 | -0.2285 | 0.2036 |
| GRA domestic | -0.0518 | -0.1997 | 0.1498 | 0.0422 | 1 | 0.2622 | 0.2216 | -0.3085 |
| GRA public | -0.1392 | -0.3303 | 0.0195 | 0.0576 | 0.2622 | 1 | -0.0544 | -0.1067 |
| WFC | 0.0989 | 0.2576 | 0.1518 | -0.2285 | 0.2216 | -0.0544 | 1 | -0.8768 |
| FWC | -0.1176 | -0.2137 | -0.2236 | 0.2036 | -0.3085 | -0.1067 | -0.8768 | 1 |

**Supplementary table 3**. Standard errors for correlation of main variables

|  | sex | age | education | employment | relationship | no. children under 13 | no. children 14-18 | family time |
| --- | --- | --- | --- | --- | --- | --- | --- | --- |
| sex | 0 | 0.0533 | 0.0713 | 0.0644 | 0.0724 | 0.0646 | 0.0383 | 0.0679 |
| age | 0.0533 | 0 | 0.0421 | 0.0532 | 0.0507 | 0.0468 | 0.0454 | 0.0470 |
| education | 0.0713 | 0.0421 | 0 | 0.0635 | 0.0668 | 0.0519 | 0.0662 | 0.0420 |
| employment | 0.0644 | 0.0532 | 0.0635 | 0 | 0.0568 | 0.0451 | 0.0510 | 0.0327 |
| relationship | 0.0724 | 0.0507 | 0.0668 | 0.0568 | 0 | 0.0436 | 0.0543 | 0.0545 |
| no. children under 13 | 0.0646 | 0.0468 | 0.0519 | 0.0451 | 0.0436 | 0 | 0.0460 | 0.0469 |
| no. children 14-18 | 0.0383 | 0.0454 | 0.0662 | 0.0510 | 0.0543 | 0.0460 | 0 | 0.0443 |
| family time | 0.0679 | 0.0470 | 0.0420 | 0.0327 | 0.0545 | 0.0469 | 0.0443 | 0 |
| pos. Affect | 0.0621 | 0.0471 | 0.0598 | 0.0532 | 0.0513 | 0.0471 | 0.0469 | 0.0461 |
| negative affect | 0.0627 | 0.0459 | 0.0559 | 0.0532 | 0.0523 | 0.0463 | 0.0464 | 0.0472 |
| life satisf. | 0.0605 | 0.0471 | 0.0599 | 0.0518 | 0.0545 | 0.0470 | 0.0471 | 0.0465 |
| stress | 0.0630 | 0.0467 | 0.0559 | 0.0534 | 0.0508 | 0.0459 | 0.0460 | 0.0444 |
| GRA domestic | 0.0623 | 0.0371 | 0.0536 | 0.0539 | 0.0380 | 0.0425 | 0.0456 | 0.0467 |
| GRA public | 0.0731 | 0.0458 | 0.0552 | 0.0587 | 0.0520 | 0.0420 | 0.0437 | 0.0471 |
| WFC | 0.0538 | 0.0471 | 0.0545 | 0.0545 | 0.0406 | 0.0458 | 0.0435 | 0.0447 |
| FWC | 0.0534 | 0.0454 | 0.0567 | 0.0529 | 0.0359 | 0.0453 | 0.0472 | 0.0421 |

**Supplementary table 4**. Standard errors for correlation of main variables (cont.)

|  | pos. Affect | negative affect | life satisf. | stress | GRA domestic | GRA public | WFC | FWC |
| --- | --- | --- | --- | --- | --- | --- | --- | --- |
| sex | 0.0621 | 0.0627 | 0.0605 | 0.0630 | 0.0623 | 0.0731 | 0.0538 | 0.0534 |
| age | 0.0471 | 0.0459 | 0.0471 | 0.0467 | 0.0371 | 0.0458 | 0.0471 | 0.0454 |
| education | 0.0598 | 0.0559 | 0.0599 | 0.0559 | 0.0536 | 0.0552 | 0.0545 | 0.0567 |
| employment | 0.0532 | 0.0532 | 0.0518 | 0.0534 | 0.0539 | 0.0587 | 0.0545 | 0.0529 |
| relationship | 0.0513 | 0.0523 | 0.0545 | 0.0508 | 0.0380 | 0.0520 | 0.0406 | 0.0359 |
| no. children under 13 | 0.0471 | 0.0463 | 0.0470 | 0.0459 | 0.0425 | 0.0420 | 0.0458 | 0.0453 |
| no. children 14-18 | 0.0469 | 0.0464 | 0.0471 | 0.0460 | 0.0456 | 0.0437 | 0.0435 | 0.0472 |
| family time | 0.0461 | 0.0472 | 0.0465 | 0.0444 | 0.0467 | 0.0471 | 0.0447 | 0.0421 |
| pos. Affect | 0 | 0.0281 | 0.0271 | 0.0299 | 0.0471 | 0.0464 | 0.0467 | 0.0465 |
| negative affect | 0.0281 | 0 | 0.0362 | 0.0322 | 0.0453 | 0.0417 | 0.0441 | 0.0450 |
| life satisf. | 0.0271 | 0.0362 | 0 | 0.0299 | 0.0461 | 0.0471 | 0.0461 | 0.0448 |
| stress | 0.0299 | 0.0322 | 0.0299 | 0 | 0.0471 | 0.0470 | 0.0447 | 0.0452 |
| GRA domestic | 0.0471 | 0.0453 | 0.0461 | 0.0471 | 0 | 0.0440 | 0.0449 | 0.0427 |
| GRA public | 0.0464 | 0.0417 | 0.0471 | 0.0470 | 0.0440 | 0 | 0.0470 | 0.0467 |
| WFC | 0.0467 | 0.0441 | 0.0461 | 0.0447 | 0.0449 | 0.0470 | 0 | 0.0112 |
| FWC | 0.0465 | 0.0450 | 0.0448 | 0.0452 | 0.0427 | 0.0467 | 0.0112 | 0 |

**Supplementary table 5**. Types of correlation tests for the correlations of main variables

|  | sex | age | education | employment | relationship | no. children under 13 | no. children 14-18 | family time |
| --- | --- | --- | --- | --- | --- | --- | --- | --- |
| sex |  | Polyserial | Polychoric | Polychoric | Polychoric | Polyserial | Polyserial | Polyserial |
| age | Polyserial |  | Polyserial | Polyserial | Polyserial | Pearson | Pearson | Pearson |
| education | Polychoric | Polyserial |  | Polychoric | Polychoric | Polyserial | Polyserial | Polyserial |
| employment | Polychoric | Polyserial | Polychoric |  | Polychoric | Polyserial | Polyserial | Polyserial |
| relationship | Polychoric | Polyserial | Polychoric | Polychoric |  | Polyserial | Polyserial | Polyserial |
| no. children under 13 | Polyserial | Pearson | Polyserial | Polyserial | Polyserial |  | Pearson | Pearson |
| no. children 14-18 | Polyserial | Pearson | Polyserial | Polyserial | Polyserial | Pearson |  | Pearson |
| family time | Polyserial | Pearson | Polyserial | Polyserial | Polyserial | Pearson | Pearson |  |
| pos. Affect | Polyserial | Pearson | Polyserial | Polyserial | Polyserial | Pearson | Pearson | Pearson |
| negative affect | Polyserial | Pearson | Polyserial | Polyserial | Polyserial | Pearson | Pearson | Pearson |
| life satisf. | Polyserial | Pearson | Polyserial | Polyserial | Polyserial | Pearson | Pearson | Pearson |
| stress | Polyserial | Pearson | Polyserial | Polyserial | Polyserial | Pearson | Pearson | Pearson |
| GRA domestic | Polyserial | Pearson | Polyserial | Polyserial | Polyserial | Pearson | Pearson | Pearson |
| GRA public | Polyserial | Pearson | Polyserial | Polyserial | Polyserial | Pearson | Pearson | Pearson |
| WFC | Polyserial | Pearson | Polyserial | Polyserial | Polyserial | Pearson | Pearson | Pearson |
| FWC | Polyserial | Pearson | Polyserial | Polyserial | Polyserial | Pearson | Pearson | Pearson |

**Supplementary table 6**. Types of correlation tests for the correlations of main variables (cont.)

|  | pos. Affect | negative affect | life satisf. | stress | GRA domestic | GRA public | WFC | FWC |
| --- | --- | --- | --- | --- | --- | --- | --- | --- |
| sex | Polyserial | Polyserial | Polyserial | Polyserial | Polyserial | Polyserial | Polyserial | Polyserial |
| age | Pearson | Pearson | Pearson | Pearson | Pearson | Pearson | Pearson | Pearson |
| education | Polyserial | Polyserial | Polyserial | Polyserial | Polyserial | Polyserial | Polyserial | Polyserial |
| employment | Polyserial | Polyserial | Polyserial | Polyserial | Polyserial | Polyserial | Polyserial | Polyserial |
| relationship | Polyserial | Polyserial | Polyserial | Polyserial | Polyserial | Polyserial | Polyserial | Polyserial |
| no. children under 13 | Pearson | Pearson | Pearson | Pearson | Pearson | Pearson | Pearson | Pearson |
| no. children 14-18 | Pearson | Pearson | Pearson | Pearson | Pearson | Pearson | Pearson | Pearson |
| family time | Pearson | Pearson | Pearson | Pearson | Pearson | Pearson | Pearson | Pearson |
| pos. Affect |  | Pearson | Pearson | Pearson | Pearson | Pearson | Pearson | Pearson |
| negative affect | Pearson |  | Pearson | Pearson | Pearson | Pearson | Pearson | Pearson |
| life satisf. | Pearson | Pearson |  | Pearson | Pearson | Pearson | Pearson | Pearson |
| stress | Pearson | Pearson | Pearson |  | Pearson | Pearson | Pearson | Pearson |
| GRA domestic | Pearson | Pearson | Pearson | Pearson |  | Pearson | Pearson | Pearson |
| GRA public | Pearson | Pearson | Pearson | Pearson | Pearson |  | Pearson | Pearson |
| WFC | Pearson | Pearson | Pearson | Pearson | Pearson | Pearson |  | Pearson |
| FWC | Pearson | Pearson | Pearson | Pearson | Pearson | Pearson | Pearson |  |

**Supplementary table 7**. Correlation effects for correlations of main variables for the female sample

|  | age | education | employment | relationship | no. children under 13 | no. children 14-18 | family time |
| --- | --- | --- | --- | --- | --- | --- | --- |
| age | 1 | -0.6468037 | 0.08413048 | -0.2550521 | -0.1004087 | 0.22804442 | -0.0828629 |
| education | -0.6468037 | 1 | 0.28477813 | 0.13593682 | 0.28297341 | -0.6443536 | 0.39786372 |
| employment | 0.08413048 | 0.28477813 | 1 | -0.2765155 | 0.38306192 | -0.4462176 | 0.62936774 |
| relationship | -0.2550521 | 0.13593682 | -0.2765155 | 1 | -0.4194157 | 0.02523873 | 0.02697098 |
| no. children under 13 | -0.1004087 | 0.28297341 | 0.38306192 | -0.4194157 | 1 | 0.1391273 | 0.08793618 |
| no. children 14-18 | 0.22804442 | -0.6443536 | -0.4462176 | 0.02523873 | 0.1391273 | 1 | -0.2721777 |
| family time | -0.0828629 | 0.39786372 | 0.62936774 | 0.02697098 | 0.08793618 | -0.2721777 | 1 |
| pos. Affect | 0.02225132 | -0.0069616 | -0.0414758 | 0.22303111 | -0.0460348 | 0.06349185 | -0.1500584 |
| negative affect | 0.17670593 | -0.1713939 | 0.10643414 | 0.1285095 | 0.13286664 | 0.15001895 | -0.0063455 |
| life satisf. | -0.0588513 | 0.1011193 | 0.08725402 | 0.07150506 | 0.06061824 | -0.0499707 | -0.1099981 |
| stress | -0.1082169 | 0.30931047 | 0.05878792 | -0.2186558 | 0.16804904 | -0.1718351 | 0.25003949 |
| GRA domestic | -0.4659228 | 0.30058383 | -0.0593087 | 0.53699842 | -0.3151065 | -0.1886656 | 0.10358425 |
| GRA public | -0.1458587 | -0.2033707 | 0.18211521 | -0.1979514 | -0.3425099 | -0.244131 | 0.0366188 |
| WFC | -0.0332725 | -0.191896 | -0.0377907 | 0.46459865 | -0.1724876 | 0.28271093 | 0.23002163 |
| FWC | 0.18843936 | -0.0060116 | -0.1003272 | -0.5383218 | 0.20402089 | -0.0014567 | -0.3233389 |

**Supplementary table 8**. Correlation effects for correlations of main variables for the female sample (cont.)

|  | pos. Affect | negative affect | life satisf. | stress | GRA domestic | GRA public | WFC | FWC |
| --- | --- | --- | --- | --- | --- | --- | --- | --- |
| age | 0.02225132 | 0.17670593 | -0.0588513 | -0.1082169 | -0.4659228 | -0.1458587 | -0.0332725 | 0.18843936 |
| education | -0.0069616 | -0.1713939 | 0.1011193 | 0.30931047 | 0.30058383 | -0.2033707 | -0.191896 | -0.0060116 |
| employment | -0.0414758 | 0.10643414 | 0.08725402 | 0.05878792 | -0.0593087 | 0.18211521 | -0.0377907 | -0.1003272 |
| relationship | 0.22303111 | 0.1285095 | 0.07150506 | -0.2186558 | 0.53699842 | -0.1979514 | 0.46459865 | -0.5383218 |
| no. children under 13 | -0.0460348 | 0.13286664 | 0.06061824 | 0.16804904 | -0.3151065 | -0.3425099 | -0.1724876 | 0.20402089 |
| no. children 14-18 | 0.06349185 | 0.15001895 | -0.0499707 | -0.1718351 | -0.1886656 | -0.244131 | 0.28271093 | -0.0014567 |
| family time | -0.1500584 | -0.0063455 | -0.1099981 | 0.25003949 | 0.10358425 | 0.0366188 | 0.23002163 | -0.3233389 |
| pos. Affect | 1 | 0.63294541 | 0.65621584 | -0.6027478 | -0.0506657 | -0.1400628 | 0.09927249 | -0.1161856 |
| negative affect | 0.63294541 | 1 | 0.47752542 | -0.569832 | -0.2024412 | -0.3304945 | 0.25735691 | -0.2173594 |
| life satisf. | 0.65621584 | 0.47752542 | 1 | -0.601776 | 0.15243088 | 0.02040615 | 0.15214903 | -0.2201832 |
| stress | -0.6027478 | -0.569832 | -0.601776 | 1 | 0.04438087 | 0.05964164 | -0.2281896 | 0.20619648 |
| GRA domestic | -0.0506657 | -0.2024412 | 0.15243088 | 0.04438087 | 1 | 0.25955523 | 0.22225473 | -0.3098599 |
| GRA public | -0.1400628 | -0.3304945 | 0.02040615 | 0.05964164 | 0.25955523 | 1 | -0.0570811 | -0.1078475 |
| WFC | 0.09927249 | 0.25735691 | 0.15214903 | -0.2281896 | 0.22225473 | -0.0570811 | 1 | -0.8740221 |
| FWC | -0.1161856 | -0.2173594 | -0.2201832 | 0.20619648 | -0.3098599 | -0.1078475 | -0.8740221 | 1 |

**Supplementary table 9**. Standard errors for correlation of main variables for the female sample

|  | age | education | employment | relationship | no. children under 13 | no. children 14-18 | family time |
| --- | --- | --- | --- | --- | --- | --- | --- |
| age | 0 | 0.04214475 | 0.05323988 | 0.05072457 | 0.0468298 | 0.04541875 | 0.04699024 |
| education | 0.04214475 | 0 | 0.06350185 | 0.06682316 | 0.05185069 | 0.06622456 | 0.0420014 |
| employment | 0.05323988 | 0.06350185 | 0 | 0.05680838 | 0.04514765 | 0.0510421 | 0.03271799 |
| relationship | 0.05072457 | 0.06682316 | 0.05680838 | 0 | 0.04360984 | 0.05434618 | 0.0545098 |
| no. children under 13 | 0.0468298 | 0.05185069 | 0.04514765 | 0.04360984 | 0 | 0.04604657 | 0.04689322 |
| no. children 14-18 | 0.04541875 | 0.06622456 | 0.0510421 | 0.05434618 | 0.04604657 | 0 | 0.04426378 |
| family time | 0.04699024 | 0.0420014 | 0.03271799 | 0.0545098 | 0.04689322 | 0.04426378 | 0 |
| pos. Affect | 0.04714469 | 0.05983779 | 0.05323866 | 0.05133536 | 0.0470704 | 0.04693395 | 0.04605297 |
| negative affect | 0.04594081 | 0.05594697 | 0.05324774 | 0.05229458 | 0.04627059 | 0.04636166 | 0.04718695 |
| life satisf. | 0.04708879 | 0.05991684 | 0.05175583 | 0.05447975 | 0.04704903 | 0.04713459 | 0.04652443 |
| stress | 0.04672893 | 0.05585175 | 0.05336284 | 0.05081819 | 0.04593161 | 0.04598528 | 0.04439473 |
| GRA domestic | 0.0370601 | 0.0535687 | 0.0538933 | 0.0379897 | 0.042489 | 0.04555773 | 0.04669574 |
| GRA public | 0.04584421 | 0.05524264 | 0.05867462 | 0.05197746 | 0.04202807 | 0.04367422 | 0.04705962 |
| WFC | 0.04713206 | 0.05451697 | 0.05447128 | 0.04062825 | 0.0457966 | 0.04346304 | 0.04468459 |
| FWC | 0.04540728 | 0.05673873 | 0.05288084 | 0.0358558 | 0.04528721 | 0.04718551 | 0.04214464 |

**Supplementary table 10**. Standard errors for correlation of main variables for the female sample (cont.)

|  | pos. Affect | negative affect | life satisf. | stress | GRA domestic | GRA public | WFC | FWC |
| --- | --- | --- | --- | --- | --- | --- | --- | --- |
| age | 0.04714469 | 0.04594081 | 0.04708879 | 0.04672893 | 0.0370601 | 0.04584421 | 0.04713206 | 0.04540728 |
| education | 0.05983779 | 0.05594697 | 0.05991684 | 0.05585175 | 0.0535687 | 0.05524264 | 0.05451697 | 0.05673873 |
| employment | 0.05323866 | 0.05324774 | 0.05175583 | 0.05336284 | 0.0538933 | 0.05867462 | 0.05447128 | 0.05288084 |
| relationship | 0.05133536 | 0.05229458 | 0.05447975 | 0.05081819 | 0.0379897 | 0.05197746 | 0.04062825 | 0.0358558 |
| no. children under 13 | 0.0470704 | 0.04627059 | 0.04704903 | 0.04593161 | 0.042489 | 0.04202807 | 0.0457966 | 0.04528721 |
| no. children 14-18 | 0.04693395 | 0.04636166 | 0.04713459 | 0.04598528 | 0.04555773 | 0.04367422 | 0.04346304 | 0.04718551 |
| family time | 0.04605297 | 0.04718695 | 0.04652443 | 0.04439473 | 0.04669574 | 0.04705962 | 0.04468459 | 0.04214464 |
| pos. Affect | 0 | 0.02808315 | 0.02714502 | 0.02992733 | 0.04706392 | 0.04635168 | 0.04672125 | 0.04653305 |
| negative affect | 0.02808315 | 0 | 0.03615438 | 0.03216336 | 0.04527968 | 0.04167406 | 0.04408556 | 0.04502975 |
| life satisf. | 0.02714502 | 0.03615438 | 0 | 0.02987903 | 0.04610859 | 0.04714044 | 0.04609187 | 0.044846 |
| stress | 0.02992733 | 0.03216336 | 0.02987903 | 0 | 0.04709763 | 0.04697157 | 0.04474663 | 0.04522966 |
| GRA domestic | 0.04706392 | 0.04527968 | 0.04610859 | 0.04709763 | 0 | 0.04397789 | 0.04486309 | 0.04265885 |
| GRA public | 0.04635168 | 0.04167406 | 0.04714044 | 0.04697157 | 0.04397789 | 0 | 0.04702943 | 0.04669249 |
| WFC | 0.04672125 | 0.04408556 | 0.04609187 | 0.04474663 | 0.04486309 | 0.04702943 | 0 | 0.01119078 |
| FWC | 0.04653305 | 0.04502975 | 0.044846 | 0.04522966 | 0.04265885 | 0.04669249 | 0.01119078 | 0 |

**Supplementary table 11**. Types of correlation tests for the correlations of main variables for the female sample

|  | age | education | employment | relationship | no. children under 13 | no. children 14-18 | family time |
| --- | --- | --- | --- | --- | --- | --- | --- |
| age |  | Polyserial | Polyserial | Polyserial | Pearson | Pearson | Pearson |
| education | Polyserial |  | Polychoric | Polychoric | Polyserial | Polyserial | Polyserial |
| employment | Polyserial | Polychoric |  | Polychoric | Polyserial | Polyserial | Polyserial |
| relationship | Polyserial | Polychoric | Polychoric |  | Polyserial | Polyserial | Polyserial |
| no. children under 13 | Pearson | Polyserial | Polyserial | Polyserial |  | Pearson | Pearson |
| no. children 14-18 | Pearson | Polyserial | Polyserial | Polyserial | Pearson |  | Pearson |
| family time | Pearson | Polyserial | Polyserial | Polyserial | Pearson | Pearson |  |
| pos. Affect | Pearson | Polyserial | Polyserial | Polyserial | Pearson | Pearson | Pearson |
| negative affect | Pearson | Polyserial | Polyserial | Polyserial | Pearson | Pearson | Pearson |
| life satisf. | Pearson | Polyserial | Polyserial | Polyserial | Pearson | Pearson | Pearson |
| stress | Pearson | Polyserial | Polyserial | Polyserial | Pearson | Pearson | Pearson |
| GRA domestic | Pearson | Polyserial | Polyserial | Polyserial | Pearson | Pearson | Pearson |
| GRA public | Pearson | Polyserial | Polyserial | Polyserial | Pearson | Pearson | Pearson |
| WFC | Pearson | Polyserial | Polyserial | Polyserial | Pearson | Pearson | Pearson |
| FWC | Pearson | Polyserial | Polyserial | Polyserial | Pearson | Pearson | Pearson |

**Supplementary table 12**. Types of correlation tests for the correlations of main variables for the male sample (cont.)

|  | pos. Affect | negative affect | life satisf. | stress | GRA domestic | GRA public | WFC | FWC |
| --- | --- | --- | --- | --- | --- | --- | --- | --- |
| age | Pearson | Pearson | Pearson | Pearson | Pearson | Pearson | Pearson | Pearson |
| education | Polyserial | Polyserial | Polyserial | Polyserial | Polyserial | Polyserial | Polyserial | Polyserial |
| employment | Polyserial | Polyserial | Polyserial | Polyserial | Polyserial | Polyserial | Polyserial | Polyserial |
| relationship | Polyserial | Polyserial | Polyserial | Polyserial | Polyserial | Polyserial | Polyserial | Polyserial |
| no. children under 13 | Pearson | Pearson | Pearson | Pearson | Pearson | Pearson | Pearson | Pearson |
| no. children 14-18 | Pearson | Pearson | Pearson | Pearson | Pearson | Pearson | Pearson | Pearson |
| family time | Pearson | Pearson | Pearson | Pearson | Pearson | Pearson | Pearson | Pearson |
| pos. Affect |  | Pearson | Pearson | Pearson | Pearson | Pearson | Pearson | Pearson |
| negative affect | Pearson |  | Pearson | Pearson | Pearson | Pearson | Pearson | Pearson |
| life satisf. | Pearson | Pearson |  | Pearson | Pearson | Pearson | Pearson | Pearson |
| stress | Pearson | Pearson | Pearson |  | Pearson | Pearson | Pearson | Pearson |
| GRA domestic | Pearson | Pearson | Pearson | Pearson |  | Pearson | Pearson | Pearson |
| GRA public | Pearson | Pearson | Pearson | Pearson | Pearson |  | Pearson | Pearson |
| WFC | Pearson | Pearson | Pearson | Pearson | Pearson | Pearson |  | Pearson |
| FWC | Pearson | Pearson | Pearson | Pearson | Pearson | Pearson | Pearson |  |

**Supplementary table 13**. Correlation effects for correlations of main variables for the male sample

|  | age | education | employment | relationship | no. children under 13 | no. children 14-18 | family time |
| --- | --- | --- | --- | --- | --- | --- | --- |
| age | 1 | -0.6468037 | 0.08413048 | -0.2550521 | -0.1004087 | 0.22804442 | -0.0828629 |
| education | -0.6468037 | 1 | 0.28477813 | 0.13593682 | 0.28297341 | -0.6443536 | 0.39786372 |
| employment | 0.08413048 | 0.28477813 | 1 | -0.2765155 | 0.38306192 | -0.4462176 | 0.62936774 |
| relationship | -0.2550521 | 0.13593682 | -0.2765155 | 1 | -0.4194157 | 0.02523873 | 0.02697098 |
| no. children under 13 | -0.1004087 | 0.28297341 | 0.38306192 | -0.4194157 | 1 | 0.1391273 | 0.08793618 |
| no. children 14-18 | 0.22804442 | -0.6443536 | -0.4462176 | 0.02523873 | 0.1391273 | 1 | -0.2721777 |
| family time | -0.0828629 | 0.39786372 | 0.62936774 | 0.02697098 | 0.08793618 | -0.2721777 | 1 |
| pos. Affect | 0.02225132 | -0.0069616 | -0.0414758 | 0.22303111 | -0.0460348 | 0.06349185 | -0.1500584 |
| negative affect | 0.17670593 | -0.1713939 | 0.10643414 | 0.1285095 | 0.13286664 | 0.15001895 | -0.0063455 |
| life satisf. | -0.0588513 | 0.1011193 | 0.08725402 | 0.07150506 | 0.06061824 | -0.0499707 | -0.1099981 |
| stress | -0.1082169 | 0.30931047 | 0.05878792 | -0.2186558 | 0.16804904 | -0.1718351 | 0.25003949 |
| GRA domestic | -0.4659228 | 0.30058383 | -0.0593087 | 0.53699842 | -0.3151065 | -0.1886656 | 0.10358425 |
| GRA public | -0.1458587 | -0.2033707 | 0.18211521 | -0.1979514 | -0.3425099 | -0.244131 | 0.0366188 |
| WFC | -0.0332725 | -0.191896 | -0.0377907 | 0.46459865 | -0.1724876 | 0.28271093 | 0.23002163 |
| FWC | 0.18843936 | -0.0060116 | -0.1003272 | -0.5383218 | 0.20402089 | -0.0014567 | -0.3233389 |

**Supplementary table 14**. Correlation effects for correlations of main variables for the male sample (cont.)

|  | pos. Affect | negative affect | life satisf. | stress | GRA domestic | GRA public | WFC | FWC |
| --- | --- | --- | --- | --- | --- | --- | --- | --- |
| age | 0.02225132 | 0.17670593 | -0.0588513 | -0.1082169 | -0.4659228 | -0.1458587 | -0.0332725 | 0.18843936 |
| education | -0.0069616 | -0.1713939 | 0.1011193 | 0.30931047 | 0.30058383 | -0.2033707 | -0.191896 | -0.0060116 |
| employment | -0.0414758 | 0.10643414 | 0.08725402 | 0.05878792 | -0.0593087 | 0.18211521 | -0.0377907 | -0.1003272 |
| relationship | 0.22303111 | 0.1285095 | 0.07150506 | -0.2186558 | 0.53699842 | -0.1979514 | 0.46459865 | -0.5383218 |
| no. children under 13 | -0.0460348 | 0.13286664 | 0.06061824 | 0.16804904 | -0.3151065 | -0.3425099 | -0.1724876 | 0.20402089 |
| no. children 14-18 | 0.06349185 | 0.15001895 | -0.0499707 | -0.1718351 | -0.1886656 | -0.244131 | 0.28271093 | -0.0014567 |
| family time | -0.1500584 | -0.0063455 | -0.1099981 | 0.25003949 | 0.10358425 | 0.0366188 | 0.23002163 | -0.3233389 |
| pos. Affect | 1 | 0.63294541 | 0.65621584 | -0.6027478 | -0.0506657 | -0.1400628 | 0.09927249 | -0.1161856 |
| negative affect | 0.63294541 | 1 | 0.47752542 | -0.569832 | -0.2024412 | -0.3304945 | 0.25735691 | -0.2173594 |
| life satisf. | 0.65621584 | 0.47752542 | 1 | -0.601776 | 0.15243088 | 0.02040615 | 0.15214903 | -0.2201832 |
| stress | -0.6027478 | -0.569832 | -0.601776 | 1 | 0.04438087 | 0.05964164 | -0.2281896 | 0.20619648 |
| GRA domestic | -0.0506657 | -0.2024412 | 0.15243088 | 0.04438087 | 1 | 0.25955523 | 0.22225473 | -0.3098599 |
| GRA public | -0.1400628 | -0.3304945 | 0.02040615 | 0.05964164 | 0.25955523 | 1 | -0.0570811 | -0.1078475 |
| WFC | 0.09927249 | 0.25735691 | 0.15214903 | -0.2281896 | 0.22225473 | -0.0570811 | 1 | -0.8740221 |
| FWC | -0.1161856 | -0.2173594 | -0.2201832 | 0.20619648 | -0.3098599 | -0.1078475 | -0.8740221 | 1 |

**Supplementary table 15**. Standard errors for correlation of main variables for the male sample

|  | age | education | employment | relationship | no. children under 13 | no. children 14-18 | family time |
| --- | --- | --- | --- | --- | --- | --- | --- |
| age | 0 | 0.04214475 | 0.05323988 | 0.05072457 | 0.0468298 | 0.04541875 | 0.04699024 |
| education | 0.04214475 | 0 | 0.06350185 | 0.06682316 | 0.05185069 | 0.06622456 | 0.0420014 |
| employment | 0.05323988 | 0.06350185 | 0 | 0.05680838 | 0.04514765 | 0.0510421 | 0.03271799 |
| relationship | 0.05072457 | 0.06682316 | 0.05680838 | 0 | 0.04360984 | 0.05434618 | 0.0545098 |
| no. children under 13 | 0.0468298 | 0.05185069 | 0.04514765 | 0.04360984 | 0 | 0.04604657 | 0.04689322 |
| no. children 14-18 | 0.04541875 | 0.06622456 | 0.0510421 | 0.05434618 | 0.04604657 | 0 | 0.04426378 |
| family time | 0.04699024 | 0.0420014 | 0.03271799 | 0.0545098 | 0.04689322 | 0.04426378 | 0 |
| pos. Affect | 0.04714469 | 0.05983779 | 0.05323866 | 0.05133536 | 0.0470704 | 0.04693395 | 0.04605297 |
| negative affect | 0.04594081 | 0.05594697 | 0.05324774 | 0.05229458 | 0.04627059 | 0.04636166 | 0.04718695 |
| life satisf. | 0.04708879 | 0.05991684 | 0.05175583 | 0.05447975 | 0.04704903 | 0.04713459 | 0.04652443 |
| stress | 0.04672893 | 0.05585175 | 0.05336284 | 0.05081819 | 0.04593161 | 0.04598528 | 0.04439473 |
| GRA domestic | 0.0370601 | 0.0535687 | 0.0538933 | 0.0379897 | 0.042489 | 0.04555773 | 0.04669574 |
| GRA public | 0.04584421 | 0.05524264 | 0.05867462 | 0.05197746 | 0.04202807 | 0.04367422 | 0.04705962 |
| WFC | 0.04713206 | 0.05451697 | 0.05447128 | 0.04062825 | 0.0457966 | 0.04346304 | 0.04468459 |
| FWC | 0.04540728 | 0.05673873 | 0.05288084 | 0.0358558 | 0.04528721 | 0.04718551 | 0.04214464 |

**Supplementary table 16**. Standard errors for correlation of main variables for the male sample (cont.)

|  | pos. Affect | negative affect | life satisf. | stress | GRA domestic | GRA public | WFC | FWC |
| --- | --- | --- | --- | --- | --- | --- | --- | --- |
| age | 0.04714469 | 0.04594081 | 0.04708879 | 0.04672893 | 0.0370601 | 0.04584421 | 0.04713206 | 0.04540728 |
| education | 0.05983779 | 0.05594697 | 0.05991684 | 0.05585175 | 0.0535687 | 0.05524264 | 0.05451697 | 0.05673873 |
| employment | 0.05323866 | 0.05324774 | 0.05175583 | 0.05336284 | 0.0538933 | 0.05867462 | 0.05447128 | 0.05288084 |
| relationship | 0.05133536 | 0.05229458 | 0.05447975 | 0.05081819 | 0.0379897 | 0.05197746 | 0.04062825 | 0.0358558 |
| no. children under 13 | 0.0470704 | 0.04627059 | 0.04704903 | 0.04593161 | 0.042489 | 0.04202807 | 0.0457966 | 0.04528721 |
| no. children 14-18 | 0.04693395 | 0.04636166 | 0.04713459 | 0.04598528 | 0.04555773 | 0.04367422 | 0.04346304 | 0.04718551 |
| family time | 0.04605297 | 0.04718695 | 0.04652443 | 0.04439473 | 0.04669574 | 0.04705962 | 0.04468459 | 0.04214464 |
| pos. Affect | 0 | 0.02808315 | 0.02714502 | 0.02992733 | 0.04706392 | 0.04635168 | 0.04672125 | 0.04653305 |
| negative affect | 0.02808315 | 0 | 0.03615438 | 0.03216336 | 0.04527968 | 0.04167406 | 0.04408556 | 0.04502975 |
| life satisf. | 0.02714502 | 0.03615438 | 0 | 0.02987903 | 0.04610859 | 0.04714044 | 0.04609187 | 0.044846 |
| stress | 0.02992733 | 0.03216336 | 0.02987903 | 0 | 0.04709763 | 0.04697157 | 0.04474663 | 0.04522966 |
| GRA domestic | 0.04706392 | 0.04527968 | 0.04610859 | 0.04709763 | 0 | 0.04397789 | 0.04486309 | 0.04265885 |
| GRA public | 0.04635168 | 0.04167406 | 0.04714044 | 0.04697157 | 0.04397789 | 0 | 0.04702943 | 0.04669249 |
| WFC | 0.04672125 | 0.04408556 | 0.04609187 | 0.04474663 | 0.04486309 | 0.04702943 | 0 | 0.01119078 |
| FWC | 0.04653305 | 0.04502975 | 0.044846 | 0.04522966 | 0.04265885 | 0.04669249 | 0.01119078 | 0 |

**Supplementary table 17**. Types of correlation tests for the correlations of main variables for the male sample

|  | age | education | employment | relationship | no. children under 13 | no. children 14-18 | family time |
| --- | --- | --- | --- | --- | --- | --- | --- |
| age |  | Polyserial | Polyserial | Polyserial | Pearson | Pearson | Pearson |
| education | Polyserial |  | Polychoric | Polychoric | Polyserial | Polyserial | Polyserial |
| employment | Polyserial | Polychoric |  | Polychoric | Polyserial | Polyserial | Polyserial |
| relationship | Polyserial | Polychoric | Polychoric |  | Polyserial | Polyserial | Polyserial |
| no. children under 13 | Pearson | Polyserial | Polyserial | Polyserial |  | Pearson | Pearson |
| no. children 14-18 | Pearson | Polyserial | Polyserial | Polyserial | Pearson |  | Pearson |
| family time | Pearson | Polyserial | Polyserial | Polyserial | Pearson | Pearson |  |
| pos. Affect | Pearson | Polyserial | Polyserial | Polyserial | Pearson | Pearson | Pearson |
| negative affect | Pearson | Polyserial | Polyserial | Polyserial | Pearson | Pearson | Pearson |
| life satisf. | Pearson | Polyserial | Polyserial | Polyserial | Pearson | Pearson | Pearson |
| stress | Pearson | Polyserial | Polyserial | Polyserial | Pearson | Pearson | Pearson |
| GRA domestic | Pearson | Polyserial | Polyserial | Polyserial | Pearson | Pearson | Pearson |
| GRA public | Pearson | Polyserial | Polyserial | Polyserial | Pearson | Pearson | Pearson |
| WFC | Pearson | Polyserial | Polyserial | Polyserial | Pearson | Pearson | Pearson |
| FWC | Pearson | Polyserial | Polyserial | Polyserial | Pearson | Pearson | Pearson |

**Supplementary table 18**. Types of correlation tests for the correlations of main variables for the male sample (cont.)

|  | pos. Affect | negative affect | life satisf. | stress | GRA domestic | GRA public | WFC | FWC |
| --- | --- | --- | --- | --- | --- | --- | --- | --- |
| age | Pearson | Pearson | Pearson | Pearson | Pearson | Pearson | Pearson | Pearson |
| education | Polyserial | Polyserial | Polyserial | Polyserial | Polyserial | Polyserial | Polyserial | Polyserial |
| employment | Polyserial | Polyserial | Polyserial | Polyserial | Polyserial | Polyserial | Polyserial | Polyserial |
| relationship | Polyserial | Polyserial | Polyserial | Polyserial | Polyserial | Polyserial | Polyserial | Polyserial |
| no. children under 13 | Pearson | Pearson | Pearson | Pearson | Pearson | Pearson | Pearson | Pearson |
| no. children 14-18 | Pearson | Pearson | Pearson | Pearson | Pearson | Pearson | Pearson | Pearson |
| family time | Pearson | Pearson | Pearson | Pearson | Pearson | Pearson | Pearson | Pearson |
| pos. Affect |  | Pearson | Pearson | Pearson | Pearson | Pearson | Pearson | Pearson |
| negative affect | Pearson |  | Pearson | Pearson | Pearson | Pearson | Pearson | Pearson |
| life satisf. | Pearson | Pearson |  | Pearson | Pearson | Pearson | Pearson | Pearson |
| stress | Pearson | Pearson | Pearson |  | Pearson | Pearson | Pearson | Pearson |
| GRA domestic | Pearson | Pearson | Pearson | Pearson |  | Pearson | Pearson | Pearson |
| GRA public | Pearson | Pearson | Pearson | Pearson | Pearson |  | Pearson | Pearson |
| WFC | Pearson | Pearson | Pearson | Pearson | Pearson | Pearson |  | Pearson |
| FWC | Pearson | Pearson | Pearson | Pearson | Pearson | Pearson | Pearson |  |
